# Doctors, Ancient and Modern

**Published:** 1882-04

**Authors:** Ausburn Towner


### DOCTORS, ANCIENT AND MODERN.

AUSBURN TOWNER.

III.

It seems easier and more reasonable, as I have observed, to believe that Esculapius was a man like other men, and not, as some have supposed, a myth. Of course he was no god. The idea of divinity attaching itself to any human personage meets with little favor in these practical days. He may have been acute, shrewd and observing, and possessed qualities not given to his cotemporaries that enabled him to become widely known as a healer of disease, or he may have been lucky in one or two instances, and thus established his reputation forever, giving his name even to the science of medicine. As in much simpler matters we know has been the case, men giving their names to a peculiar kind of coat, like the Mackintosh or Raglan, or to an act, like Burking or Boycott-ing. The whole medical faculty was at one time in the ancient days known as Asclepiades, or descendants of Esculapius, and all the medical knowledge of the time, not overmuch to be sure, rested with them. It was a sort of a family inheritance, no one outside for many years being permitted to be initiated into the mysteries of the healing art.

The close-corporation character of the members of the medical profession is no new thing, it will be seen. It has existed ever since there was a faculty, and seems to be inherent in it, regarding with suspicion all those, no matter how successful they may be, unless they are regularly appointed to practice by certain constituted authorities. Yet often these very men are the ones who elevate the character of the profession and bring it into high repute.

As an instance, there was a Greek physician who came to Rome a little more than one hundred years before Christ was born. His name was Asclepiades, but whether he had assumed this, or was really a descendant of Esculapius, is not clearly known. He was a great friend of the orator Cicero. The practice of medicine in Rome at that time was not in very good repute. There was a doctor there called Archagathus, who had had some success, but his remedies, his patients thought and said, were worse than their diseases. Asclepiades was a rhetorician, and without much study conceived the idea that he was a physician as well. The regulars looked askant at him, but his reputation was made almost in an instant by an incident. He was passing one day near a funeral train, and observed that the body being conveyed to the funeral pile showed some signs of life. He insisted upon it that the body was not dead; made the train stop, and, applying some active measures, resuscitated it, much to the astonishment of the bystanders, who proclaimed it abroad that he had restored to life a dead man. His fortune was therefore made. Besides, he was a very handsome man and had plenty of assurance. He took the opposite course with his patients to that adopted by the regular practitioners, giving pleasant remedies and rejecting the tortures that were in vogue. The simplicity of his treatment, as well as the slight knowledge of remedies, may be judged from the fact that he admitted only five means of cure: dieting, occasional abstinence from wine, frictions, exercise on foot, and the being carried in litters, none of them very unpleasant. The third one may be the original of what is now known as the movement cure, or the laying on of hands, adopted with marvelous success by some physicians in our day, who are not "regular."

Asclepiades merits mention from this other one circumstance, that of all the physicians of his time, and there must have been in a city like Rome, with its estimated population of between five and six millions of inhabitants, multitudes of them, his name is the only one that has come down to us, and he is called by Pliny a "successful charlatan," who only flattered the whims of his patients. If he was, it should be added that he succeeded in curing them too.

While speaking of charlatans, or presumed impostors of that age, one cannot help recalling the very peculiar career of Apollonius, of Tyana. It is peculiar in itself, but attains a singular attraction from the fact that it was run at about the same time as that of our Saviour, and in the particular of healing of diseases is very like it. Of the existence of this strange man there is no doubt, for temples were erected to his memory; many of his works are preserved, and his history was written out by cotemporaries. He was born at about the same time that Christ was born, and similar announcements were said to have been made to his mother before his birth as were made to the Virgin Mary. His family was a wealthy one, and he received an education as thorough and perfect as could be given in those days. His early years were spent in a temple of Esculapius at Ægeæ, where, the priests finding him docile and talented, initiated him into the mysteries of the healing art. He then spent five years without exercising the faculty of speech. Then he traveled through distant countries, pretending that, although he had never learned them, he was skilled in all languages. A natural inquiry might be made here, what was he doing all of those five years when he was silent? He pretended also to understand the language of beasts and birds! For some of his cures in some of the countries through which he passed, he was worshipped as a god. Many miracles of his performance are gravely related by his historians, among them raising the dead, casting out demons, and foretelling events. That kind of business seems to have been very common just at that period of the world's history. Apollonius became the friend of one or two of the emperors of Rome. He lived to be ninety-seven years of age. At one of his last public appearance a strange thing happened. He lived at Ephesus, many miles from Rome, and one day he was engaged in a public disputation. Suddenly, in the midst of a sentence, he stopped and exclaimed: "The emperor is dead; he is killed this very hour." And it so turned out to be. In the absence of telegraphs, it can only be conjectured that he knew the fact he proclaimed from a previous knowledge that some conspirators would make such an attempt at that very hour. The exclamation of a Nihilist in Paris when the late emperor of Russia was blown to pieces, that "the tyrant is slain," argued no very great supernatural power, and if the fact had not been as it was, neither exclamation would ever have been heard of.

The claim of Apollonius to our notice arises from the disposition existing in his time, and as Christianity began to gain ground, to assimilate his character and merits with those of the Divine Founder of our religion.

Very soon after this and a disciple of one of the friends of Apollonius, there appeared another unmistakeable medical impostor, Alexander of Abonoteichos. He came of a wretched family, but was a strangely beautiful youth. He was a confidence man of the highest order. At his wits' end to obtain a means of livelihood, he pretended that Esculapius had paid him a visit and had endowed him with all sorts of medical knowledge. Then he juggled a live serpent out of an egg, and on a larger serpent put the head of a man, and spoke or prescribed through that! He succeeded so well in fooling the citizens of Paphlagonia, where he took up his residence, that he was enabled to build a costly temple there to Esculapius, where it is certain that, undoubted charlatan as he was, he performed many singular cures, having at one time one of the emperors of Rome as one of his patients. He died when he had arrived at the age of seventy years, just as that emperor was having a gold medal struck in his honor as a god!

Such personages as this Apollonius and Alexander may seem hardly to merit a place among the doctors of old times, as having attained no prominence from any permanent good that they did, but of the benefit that they were to those persons of their own period there is no doubt. Whatever may have been the means they employed, they went about healing the sick, and they had the common people always on their side, too. Centuries would seem to have made but little difference in this phase of human nature. Every century, every generation, every decade, every community almost, sees its Apollonius or Alexander, in greater or lesser degree, and the success they achieve bewilders one to determine which is the genuine and which the charlatan. It is small comfort to a dying man, or to the friends he leaves behind, to know that he passes away under the most scientific style of treatment, when they have reason to believe that his life could have been saved by another treatment that may not be so rigidly scientific or regular. There are numerous instances in this city where the old-school guessers after a cure have given up patients as beyond the help of medical knowledge, and other persons have brought them back to life and health. When we see cases of this kind in our times and midst, we can afford to be a little charitable to those ancient people, not so well informed and practical as we, who, for similar reasons, attributed to healers like Esculapius, Apollonius of Tyana, and Alexander of Abonoteichos, a divine origin and a divine nature.

